# Scapular osteochondroma with pseudo-winging: a report of two cases

**DOI:** 10.1093/jscr/rjaf117

**Published:** 2025-03-07

**Authors:** Konstantina Pouli, Eleni Pappa, Anastasia Pilichou, Panagiotis Krallis, John Anastasopoulos

**Affiliations:** 2^nd^ Department of Orthopaedic Surgery and Traumatology, Aghia Sophia Pediatric General Hospital, Thivon 3 Street, Athens 15772, Greece; 2^nd^ Department of Orthopaedic Surgery and Traumatology, Aghia Sophia Pediatric General Hospital, Thivon 3 Street, Athens 15772, Greece; 2^nd^ Department of Orthopaedic Surgery and Traumatology, Aghia Sophia Pediatric General Hospital, Thivon 3 Street, Athens 15772, Greece; 2^nd^ Department of Orthopaedic Surgery and Traumatology, Aghia Sophia Pediatric General Hospital, Thivon 3 Street, Athens 15772, Greece; 2^nd^ Department of Orthopaedic Surgery and Traumatology, Aghia Sophia Pediatric General Hospital, Thivon 3 Street, Athens 15772, Greece

**Keywords:** winging of scapula, osteochondroma, pseudo-winging, scapula, ventral osteochondroma

## Abstract

Scapular pseudo-winging caused by ventral osteochondromas is a condition that has been rarely reported in the current literature. This manuscript reports two cases of scapular ventral osteochondromas in pediatric patients. Two female pediatric patients presented at our department with swelling mass of the scapula as well as pseudo-winging. The physical examination revealed a bony mass stemming from the body of the scapula. The diagnostic imaging included radiographs and CT, depicting the presence of ventral osteochondromas located on the body of the scapula in both patients. Surgical excision was performed leading to excellent post-operative results with complete resolution of deformity and symptoms. This case report highlights the role of ventral osteochondromas as a reason for scapular pseudo-winging. Clinical suspicion should be raised among orthopaedic surgeons for proper recognition and targeted management of scapular pseudo-winging cases caused by ventral osteochondromas.

## Introduction

Winged scapula is the condition of increased prominence of the medial border of the scapula due to long thoracic nerve disfunction, causing serratus anterior muscle impairment. Pseudo-winging of the scapula is defined as the prominence of the medial border of the scapula due to other causes than serratus anterior muscle palsy. In 1867, Demarquay first described the necropsy findings of an osteochondroma arising from the deep surface of the scapula [1]. Osteochondromas constitute 10%–15% of all bone tumors and 30%–50% of benign bone tumors, representing the most common benign tumors [2]. They are common in childhood and adolescence and stop growing after skeletal maturity [3]. It is more a developmental physeal growth defect than a tumor and usually it occurs in the metaphysis of long bones with the distal femur, proximal humerus and proximal tibia constituting 90% of all occurrences. It is unusual in flat bones with a 3%–4.5% involving scapula [3, 4]. However, this is the most common benign primary bone tumor affecting scapula [2]. When it arises from scapula usually it is ventral. However, literature is scanty about ventral scapular osteochondroma (VSO) [5]. These tumors (osteochondromas) are usually solitary (90%) but can be multiple in hereditary multiple exostoses (10%) [2]. Scapular Osteochondromas have been mentioned in the current literature as a common cause of scapular pseudo-winging, as they cause swelling in the anterior surface of the scapula, which is the main differentiating factor from the scapular winging which is caused by the long thoracic nerve dysfunction leading to serratus anterior muscle paralysis [1].

Hereditary multiple exostoses (HME) is a rare genetic disorder, inherited in an autosomal dominant manner, characterized by growths of multiple osteochondromas arising from any bone which grows from endochondral ossification excluding facial bones. In HME the metaphyseal segments of long bones are mostly affected but scapulae can be affected too, having aesthetic impact on patients, even from the childhood. Surgical resection is the treatment of choice when there are symptomatic exostoses or a strong suspicion for malignant transformation, even though the latter is characterized by low frequency (2%–4%) [6].

This case report describes two cases with a large ventral scapular osteochondroma causing pseudo-winging and snapping scapula syndrome, which were treated with surgical excision at our department.

## Case history

A 14-year-old and a 9-year-old right-hand dominant Caucasian female patients, both presented to our orthopaedic clinic with a chronic noticeable deformity of the left scapular region which progressed gradually. The otherwise healthy girls had known history of HME. They did not complain about pain or tenderness and there was no history of trauma. The 14-year-old girl had no restriction of shoulder movement while the 9-year-old girl had little restriction of flexion of the left shoulder. Birth history was insignificant, with both patients having an unremarkable medical history record.

**Figure 1:**
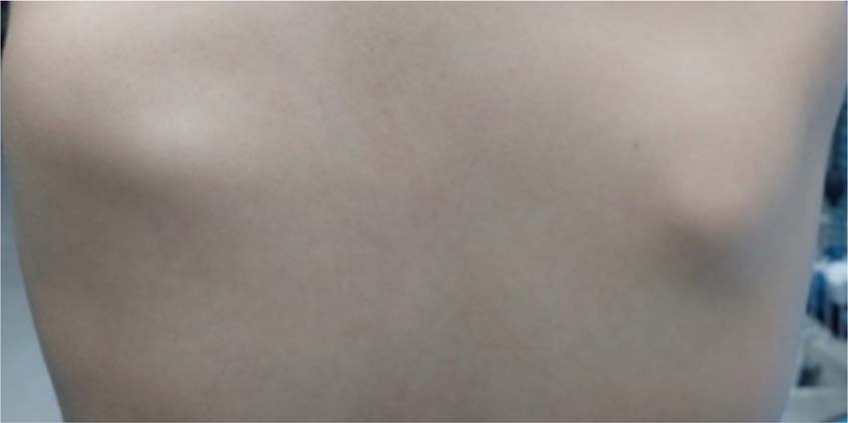
Preoperative clinical presentation showing pseudo-winging of left scapula.

On clinical examination there was a distinct asymmetry in both patients, with the left side of the thoracic cage being prominent at rest (Fig. 1). The pseudo-winging of the medial edge of the left scapula was visible at rest and did not increase by pressing the extended upper limb against the wall. There was no muscle weakness or atrophy, and all neurovascular examinations of cervical spine and upper limbs were normal. In passive and active movements of left shoulder there was a painless, non-tender snapping of the scapula but with full range of motion of the limb in the 14-year old patient. No snapping of left scapula reported of the younger girl. No functional limitations were signed in both patients.

## Case diagnosis

**Figure 2:**
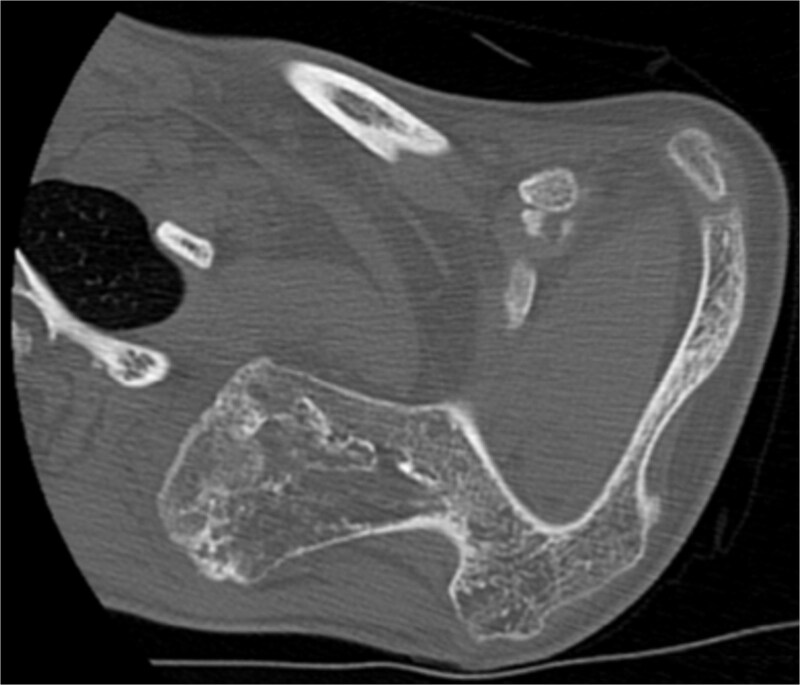
CT of the 14-year-old patient showing the largest lesion arising ventrally from the superior angle of left scapula in axial plane.

**Figure 3:**
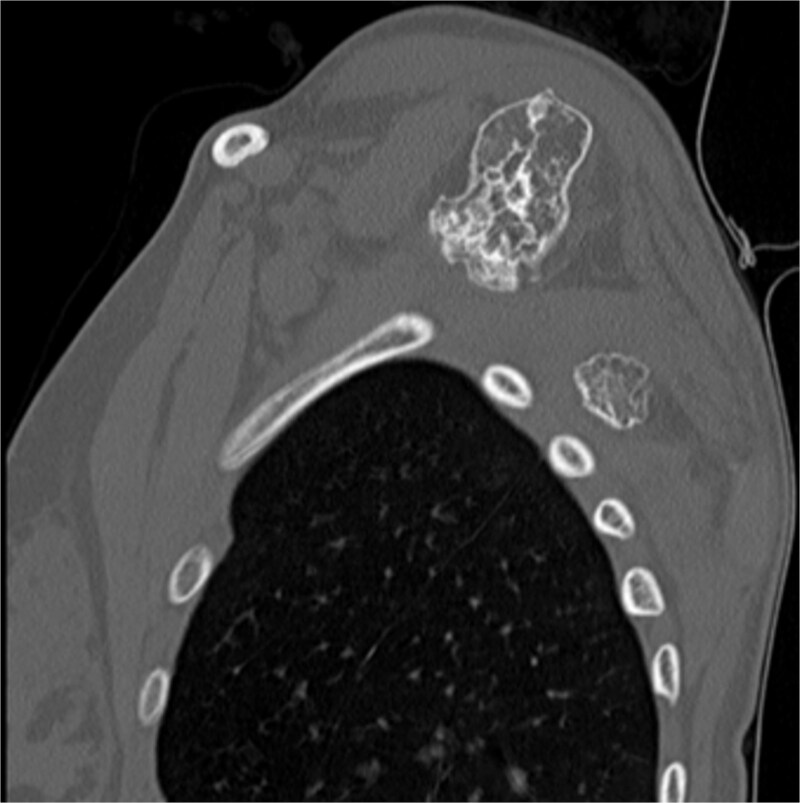
CT of the 14-year-old patient showing both lesions in sagittal plane.

The imaging investigation was initiated by our department, as we were clinically suspicious of the presence of osteochondromas, due to the history of HME in both patients. Both patients were subscribed an anteroposterior X-ray of left shoulder and a CT scan for pre-operative planning. Also, radiographic evaluation of both knees and left forearm were taken and revealed multiple exostoses as a part of the HME follow-up that both girls underwent. The CT scan of the 14 year old patient demonstrated three bony lesions arising from the ventral aspect of left scapula. The largest one extruded from superior angle heading medially to the vertebral bodies. The other two smaller arose one from the lateral angle and one from the inferior angle. The CT scan of the 9 year old patient revealed one bony lesion arising ventrally from the superior angle of the left scapula heading medially as well (Figs 2 and 3). The diagnosis of VSO was made in both cases.

## Case treatment

**Figure 4:**
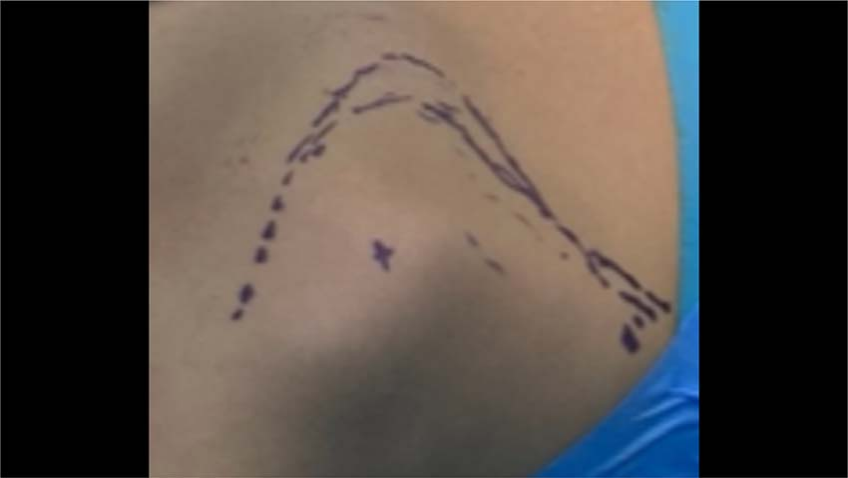
Intraoperative images of the marking of the modified Judet approach at the older patient.

**Figure 5:**
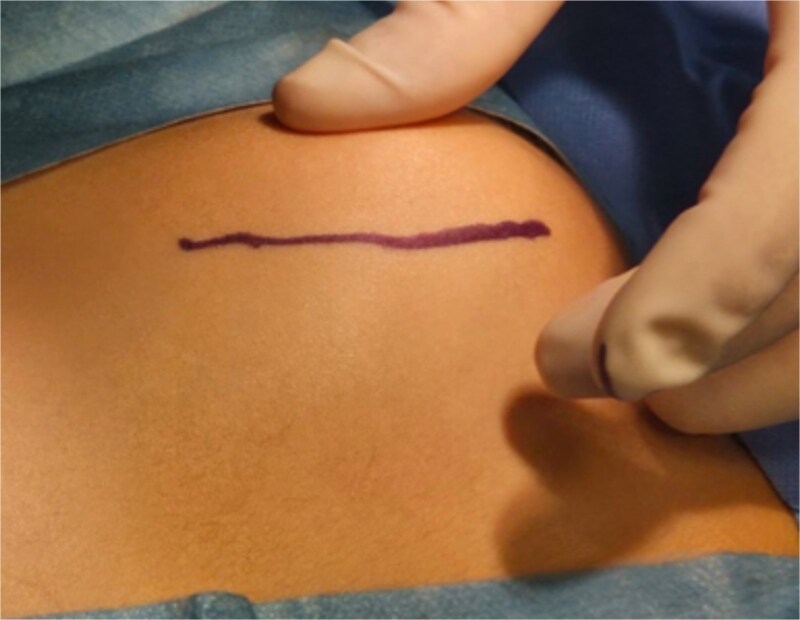
Intraoperative images of the marking of the modified Judet approach at the younger patient.

**Figure 6:**
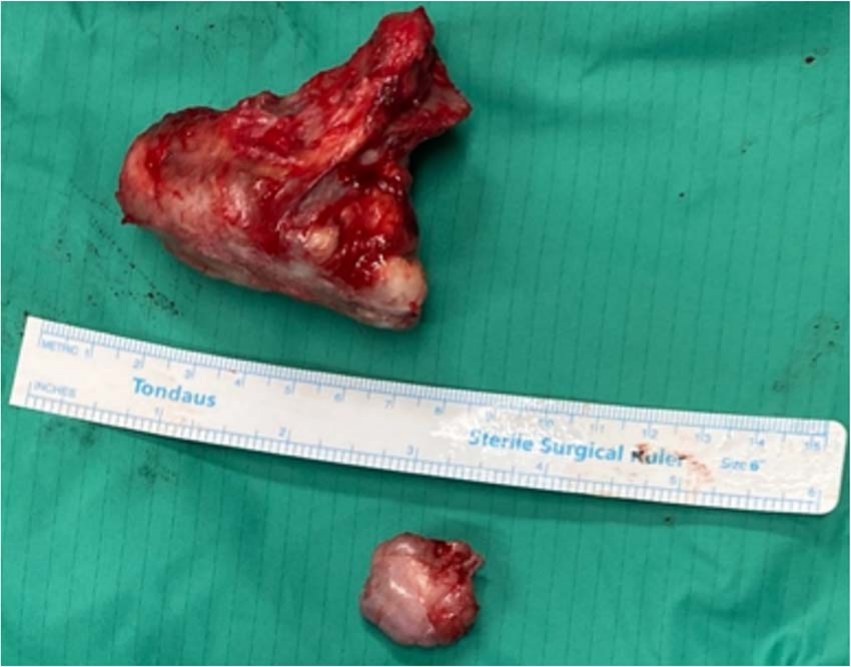
The gross specimens of the ventral scapular osteochondromas of the older patient after en bloc excision.

**Figure 7:**
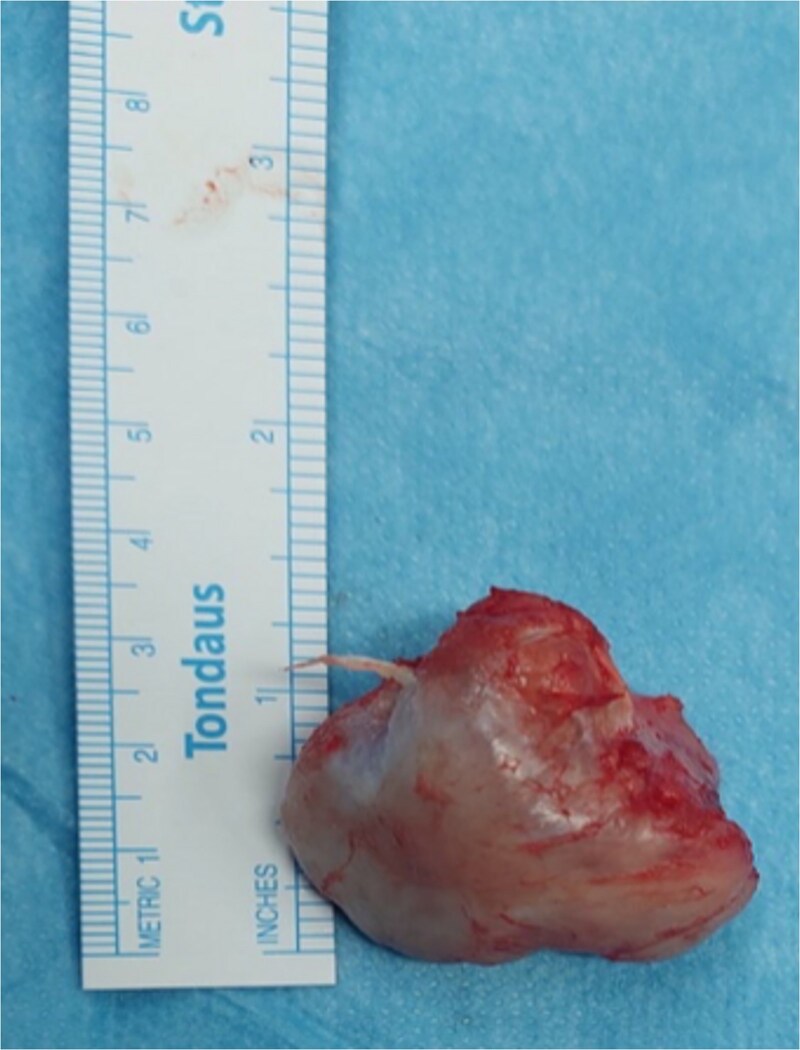
The gross specimen of the ventral scapular osteochondroma of the younger patient after en bloc excision.

Excision of the lesions was decided for both mechanical and cosmetic reasons. Wide, open excision under general anesthesia was the treatment of choice. Modified Judet approach was performed in prone position (Figs 4 and 5). A longitudinal incision along the medial border of left scapula was used. Trapezius and part of the levator scapulae were incised while rhomboids were retracted. The mass was resected en bloc, without any adhesions. Afterwards, we extended the incision in line with the tip of scapula, and we moved the left arm to neutral anatomical position so that the scapula would rotate downwards and make the mass attached to the inferior angle easier to approach and excise en bloc as well leaving the rhomboids untouched. There was no need to excise the third small lesion. For the 9-year-old patient the left arm was in neutral position from the beginning, the lesion was easily approached and excised en bloc as well. Then, after proper haemostasis, we sutured the levator scapulae fibers, and we closed by suturing in layers. For the first patient the largest lesion was pedunculated and measured ~6.5 × 5 cm and the second one was round and measured ~2 × 2 cm (Fig. 6), while for the second patient the unique lesion was pedunculated and measured ~3 × 3 cm (Fig. 7). All were sent for histological examination.

### Follow-up

**Figure 8:**
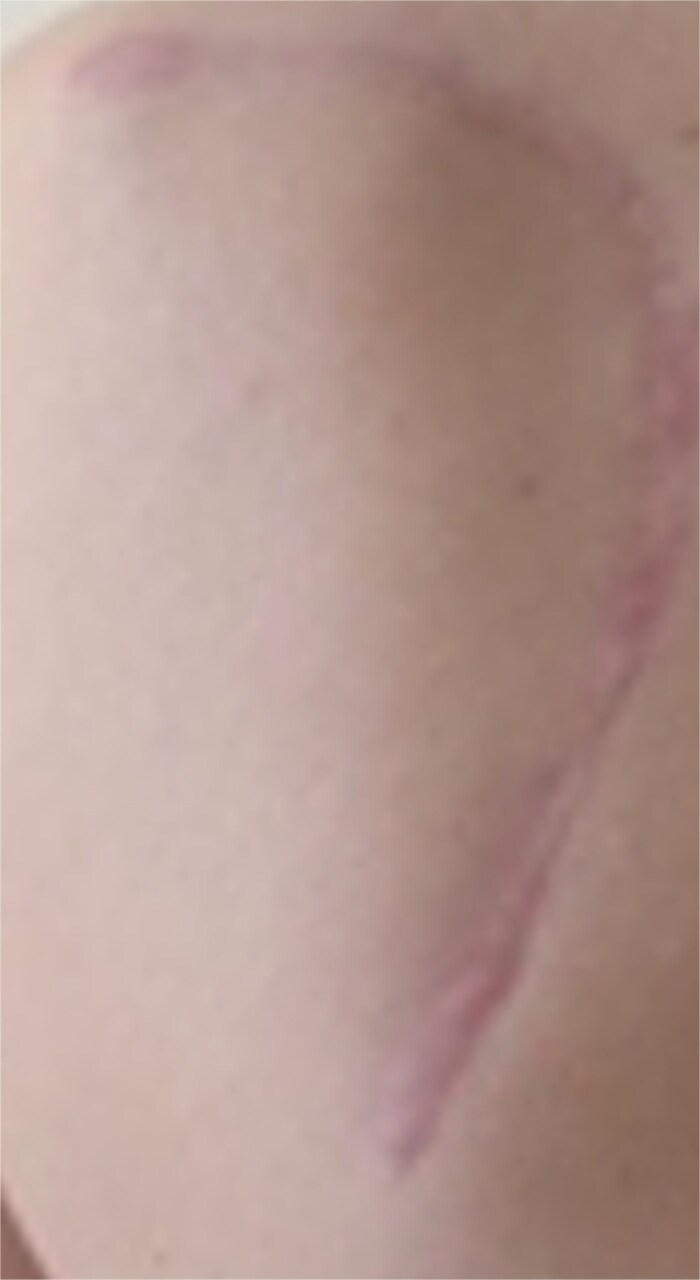
Postoperative clinical assessment without scapular prominence.

The post-operative course was uneventful for both girls (Fig. 8). The VAS score was used in both patients for post-operative pain which was signed as no pain, however, the rest exostoses in both patients were not surgically removed as they did not cause any discomfort. In 1 year follow-up both patients are participating fully in their every day activities without any recurrence of their scapular lesions.

## Discussion

Tumors involving the ventral surface of the scapula are rare, commonly generating pseudo-winging, snapping and at times may be painful. Excision of osteochondromas that arise from the ventral aspect of scapula is challenging due to the numerous muscle origins and attachments, thereby causing extensive soft tissue dissection and multiple muscular detachments. This necessitates the need of a minimally invasive and muscle-sparing procedure [7].

Various surgical approaches have been described to excise ventral scapular osteochondromas, however the modified Judet approach is a safe method for en bloc excision without muscle interference and adhesions generation. The longitudinal incision used is easily extensile and the approach gives satisfactory operative exposure for such cases. Furthermore, it does minimal trauma to the rotator cuff musculature, has lesser soft tissue damage and contaminant blood loss and protects major neurologic structures, such as suprascapular nerve superiorly and axillary nerve laterally. Two last significant assets that support its use are the quick and complete recovery of shoulder function and good cosmetic results.

Such case reports are quite rare in the current literature. Das *et al.* in their case report in 2023, reported excellent disabilities of the arm, shoulder and hand (DASH) scores after the surgical excision of similar scapula osteochondroma, while Clarke *et al* also achieved similar satisfying post-operative functional results after scapular osteochondroma excision via the modified Judet approach [8–11]. Even minimally invasive surgical techniques have been used regarding the surgical excision of ventral scapular osteochondromas, where pre-operative 3D CT reconstruction has also been used for the precise localization of the osteochondroma, as reported by Wu et al in their case report in 2024. [12]

Despite the ventral location of the scapular osteochondromas, the dorsal presence has also been highlighted in the current literature. Khan *et al.* and Jangir *et al.* in their case reports in 2024 managed surgically dorsal osteochondromas in their patients having excellent post-operative results. What is to be mentioned is that dorsal osteochondromas of the scapula are less reported in the current literature in comparison with the ventral ones [13, 14].

What is to be highlighted is that osteochondromas are characterized by continuous growing until the skeletal maturity is achieved, so orthopaedic surgeons should always be aware of likely malignant transformation if the mass growth insists, especially during the entity of MHE [15–17]. However, in our case series the possibility of malignancy was low as assumed by the radiologic depictment of cartilage cap of all the lesions. Nevertheless, the mass removal was made both for mechanical and pathologic assessment.

## Conclusion

Scapular osteochondromas are rare in the current literature. The natural history is continuous growth until skeletal maturation. By reporting the case report above, we aim to increase the clinical suspicion of the orthopaedic surgeons for any atypical sites of osteochondroma, especially the scapula, as well as their management by which excellent functional results are achieved by simple surgical excision. The ventral osteochondromas of the scapula seem to be more common in the pediatric population, however the Judet approach seems to be a safe surgical approach which guarantees an excellent post-operative result as in our cases above.
